# S-endoglin expression is induced in hyperoxia and contributes to altered pulmonary angiogenesis in bronchopulmonary dysplasia development

**DOI:** 10.1038/s41598-020-59928-x

**Published:** 2020-02-20

**Authors:** Yeongseok Lee, Juyoung Lee, Soo Kyung Nam, Yong Hoon Jun

**Affiliations:** 1 Inha University Hospital, Department of Paediatrics, Incheon, 22332 Korea; 2 Inha University College of Medicine, Department of Paediatrics, Incheon, 22212 Korea

**Keywords:** Neonatology, Paediatric research, Molecular medicine

## Abstract

Altered pulmonary angiogenesis contributes to disrupted alveolarization, which is the main characteristic of bronchopulmonary dysplasia (BPD). Transforming growth factor β (TGFβ) plays an important role during lung vascular development, and recent studies have demonstrated that endoglin is engaged in the modulation of TGFβ downstream signalling. Although there are two different isoforms of endoglin, L- and S-endoglin, little is known about the effect of S-endoglin in developing lungs. We analysed the expression of both L- and S-endoglin in the lung vasculature and its contribution to TGFβ-activin-like kinase (ALK)-Smad signalling with respect to BPD development. Hyperoxia impaired pulmonary angiogenesis accompanied by alveolar simplification in neonatal mouse lungs. S-endoglin, phosphorylated Smad2/3 and connective tissue growth factor levels were significantly increased in hyperoxia-exposed mice, while L-endoglin, phosphor-Smad1/5 and platelet-endothelial cell adhesion molecule-1 levels were significantly decreased. Hyperoxia suppressed the tubular growth of human pulmonary microvascular endothelial cells (ECs), and the selective inhibition of ALK5 signalling restored tubular growth. These results indicate that hyperoxia alters the balance in two isoforms of endoglin towards increased S-endoglin and that S-endoglin attenuates TGFβ-ALK1-Smad1/5 signalling but stimulates TGFβ-ALK5-Smad2/3 signalling in pulmonary ECs, which may lead to impaired pulmonary angiogenesis in developing lungs.

## Introduction

The main pathologic finding of bronchopulmonary dysplasia (BPD) in the postsurfactant era is an alveolar arrest resulting in alveolar simplification (the development of fewer but larger alveoli with decreased septation) and decreased density of capillary beds^1–6^. Recent studies have revealed that impaired angiogenesis may lead to decreased numbers of alveoli; therefore, enhancement of normal angiogenesis could promote alveolarization in preterm infants^1,3,5,7–10^. Indeed, evidence suggests that pulmonary vessels actively promote alveolar development in the distal saccules and that dysmorphic changes in the pulmonary vasculature contribute to alveolar hypoplasia in animal models of BPD^1,3–7,11,12^. Postmortem studies on infants who died after prolonged mechanical ventilation have also quantified abnormal pulmonary microvascular growth^13,14^.

Transforming growth factor β (TGFβ) plays a pivotal role during lung development and angiogenesis through the regulation of endothelial cell (EC) growth, differentiation, migration, senescence, and extracellular matrix (ECM) production^15–19^. TGFβ-mediated signalling is initiated by binding to a TGFβ specific membrane receptor complex in ECs that contains 3 types of receptors: type I and type II serine/threonine kinase receptors and a co-receptor or TGFβ type III receptor named endoglin, also known as CD105. These receptors propagate the signal to the downstream nuclear effectors, Smads, by phosphorylation^16,18,20–22^. Phosphorylated Smads bind to the common Smad4 protein, translocate into the nucleus and regulate the transcription of their target genes. Depending on the TGFβ type I receptor involved, the signal is propagated to two different Smad protein subfamilies with specificities for activin-like kinase 1 (ALK1) phosphorylating Smad1/5 or ALK5 phosphorylating Smad2/3^21–26^. It has also been reported that vascular endothelial growth factor (VEGF) expression increases through enhancement of the TGFβ-ALK1-Smad1/5 pathway, while connective tissue growth factor (CTGF) expression increases through enhancement of the TGFβ-ALK5-Smad2/3 pathway^27–31^.

Several independent findings have demonstrated that endoglin is engaged in TGFβ receptor complex formation and the modulation of downstream signalling^32–35^. The expression of two alternatively spliced isoforms, long (L-) endoglin and short (S-) endoglin, has been demonstrated in human and mouse lung tissues *in vivo*^33,34,36,37^. L-endoglin is the predominant isoform, and its pre-mRNA can be alternatively spliced by intron retention, producing the less abundant form, S-endoglin^36,37^. Evidence indicates that L-endoglin, the predominant isoform in ECs, promotes EC proliferation via TGFβ-ALK1 signalling, whereas S-endoglin acts as an antagonist of L-endoglin via activation of the TGFβ-ALK5 pathway^32,33,36,37^.

To date, most studies published on endoglin have focused on L-endoglin. It has been found that L-endoglin enhances ALK1-Smad1/5 signalling in ECs, leading to proliferation and migration, which are the main characteristics of the activation phase of angiogenesis^15,18,20,22,38–43^. However, a role of S-endoglin during endothelial senescence was recently described^36,37^. The S-endoglin:L-endoglin ratio increases during the senescence of ECs *in vitro* as well as during ageing in vascularized tissues, and the switch from L-endoglin to S-endoglin affects TGFβ-mediated cell signalling towards promoting the ALK5-Smad2/3 pathway instead of the ALK1-Smad1/5 pathway^23,37,43–45^.

Although the role of S-endoglin in TGFβ signalling was recently addressed in mature systemic ECs, little is known about the expression and role of S-endoglin in immature pulmonary ECs and their impacts on BPD-associated dysangiogenesis. In the present study, we analysed the expression of both L-endoglin and S-endoglin in the neonatal lung vasculature and its contribution to TGFβ-ALK-Smad signalling with respect to BPD development.

## Results

### Hyperoxia impairs alveolar and pulmonary vascular development in neonatal mice

**Figure 1:**
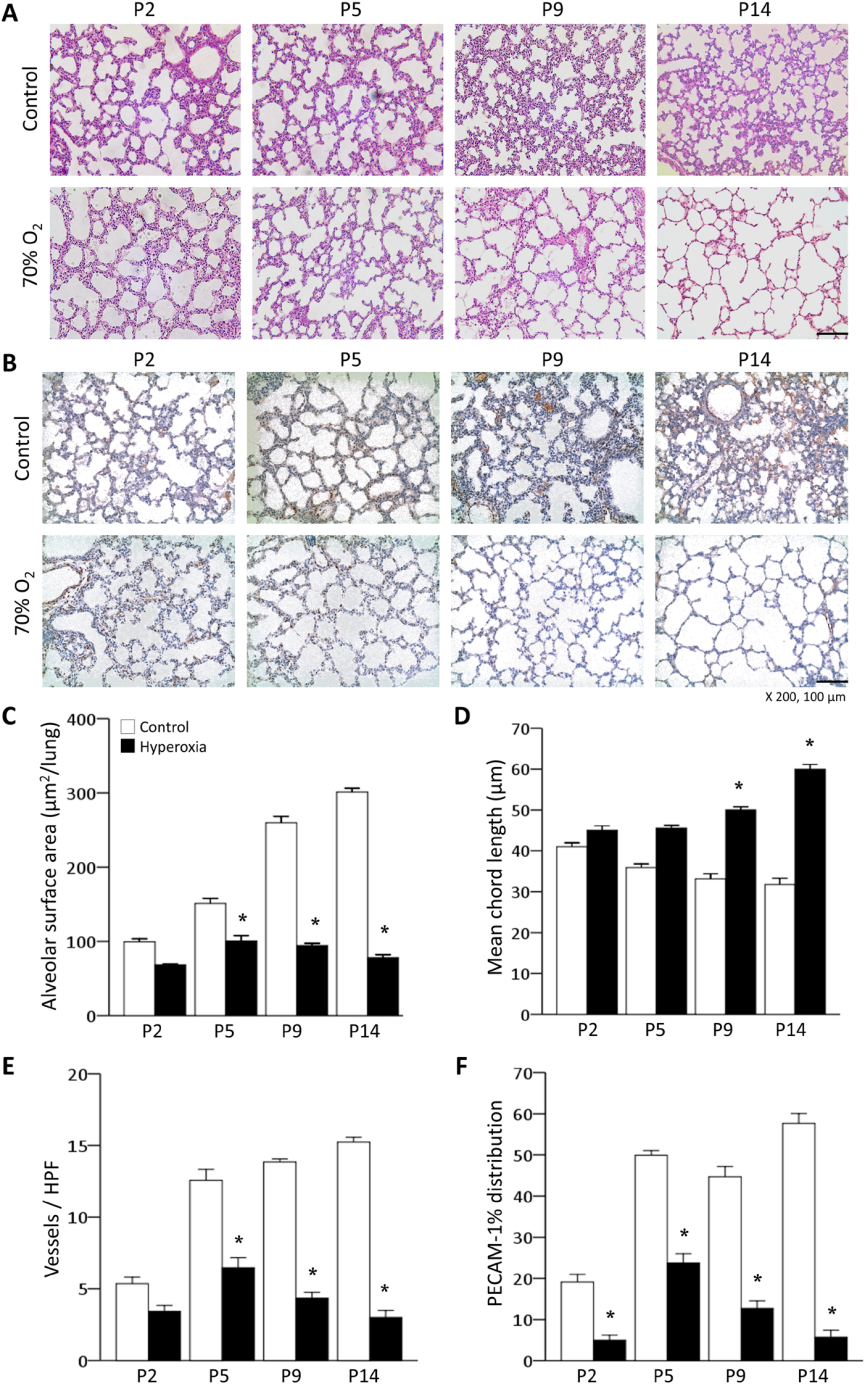
Effect of hyperoxia on alveolar and pulmonary vascular development. (**A**) Representative light microscopic images of mouse lungs. Apparently thinned septal walls and markedly large and simple airspaces were observed in the hyperoxia group compared with the control group. H&E staining: original magnification 200×. Bars, 100 µm. (**B**) Representative light microscopic images of PECAM-1 immunohistochemistry of mouse lungs. Markedly fewer blood vessels and decreased PECAM-1 staining were observed in the hyperoxia group compared with the control group. PECAM-1 staining was visualized with a DAB reaction (brown colour). Light H&E staining was used as a counterstain: original magnification 200×. Bars, 100 µm. (**C**) The alveolar SA was smaller in the hyperoxia group than in the control group; it increased as the lungs developed in the control group but not in the hyperoxia group. (**D**) The mean cord length, indicating the average alveolar size, was greater in the hyperoxia group than in the control group; it decreased as the lungs developed in the control group, but not in the hyperoxia group. (**E**) The number of blood vessels per HPF was significantly lower in the hyperoxia group than in the control group. (**F**) Immunohistochemical staining for PECAM-1 was significantly decreased in the hyperoxia group compared with the control group. (**C–F**) White bars denote the control group, and black bars denote the hyperoxia group. The data are expressed as the mean ± SEM (n = 5–7 in each group). Significant differences (*p* < 0.05) compared with the control group are marked by *.

The unexposed lungs of control mice showed normal alveolarization, indicated by small and complex distal air spaces. However, the hyperoxia group showed impaired alveolarization, with fewer, larger and simpler alveoli on P14 (Fig. 1A). The hyperoxia group had a significantly smaller alveolar surface area (SA) and a significantly longer mean chord length than the control group on P14 (Fig. 1C,D). Pulmonary vessel staining with platelet-endothelial cell adhesion molecule (PECAM)-1 was significantly lower in the hyperoxia group than in the control group during the entire experimental period (Fig. 1B,E,F). Quantification of these parameters indicated that exposure of newborn mice to hyperoxia during the early alveolar period resulted in decreased sprouting angiogenesis and impaired alveolarization.

### Hyperoxia attenuates the expression of L-endoglin but promotes the expression of S-endoglin

**Figure 2:**
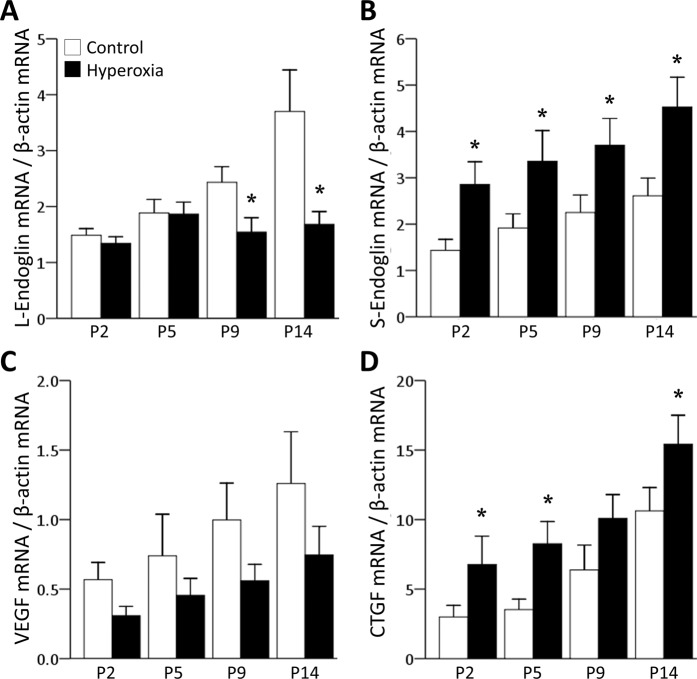
Expression of L-endoglin, S-endoglin, VEGF and CTGF mRNAs in hyperoxia. (**A**) Compared with the control group, the mRNA levels of L-endoglin were significantly decreased at P9 and P14 in the hyperoxia group. (**B**) Those of S-endoglin were significantly increased in the hyperoxia group over the entire experimental period. (**C**) VEGF showed no statistically significant difference between the control and hyperoxia groups. (**D**) The mRNA levels of CTGF were significantly increased at P2, P5 and P14 in the hyperoxia group. White bars denote the control group, and black bars denote the hyperoxia group. The data are expressed as the mean ± SEM (n = 4–6 in each group). Groups of data were compared with the corresponding time point by the Mann-Whitney U test. Significant differences (*p* < 0.05) compared with the control group are marked by *.

The level of L-endoglin mRNA was significantly decreased at P9 and P14 in the hyperoxia group, whereas S-endoglin mRNA expression was significantly increased over the entire experimental period in the hyperoxia group compared to the control group (Fig. 2A,B). Although VEGF mRNA expression was reduced in the hyperoxia group, the difference was not significant (Fig. 2C). CTGF mRNA expression was higher in the hyperoxia group than in the control group (Fig. 2D). As CTGF seems to be a key mediator of the profibrotic effect of TGFβ^36,46^, we assessed whether hyperoxia could modify CTGF expression via L- or S-endoglin. Hyperoxia in newborn mice attenuated the mRNA expression of L-endoglin but promoted the mRNA expression of S-endoglin. These results indicate that hyperoxia enhances switching from L-endoglin to S-endoglin, leading to attenuation of the active proliferation of lung and pulmonary vessels as well as promotion of pulmonary fibrosis.

### Hyperoxia alters the production of L- and S-endoglin and phosphorylated Smad proteins in neonatal mice

**Figure 3:**
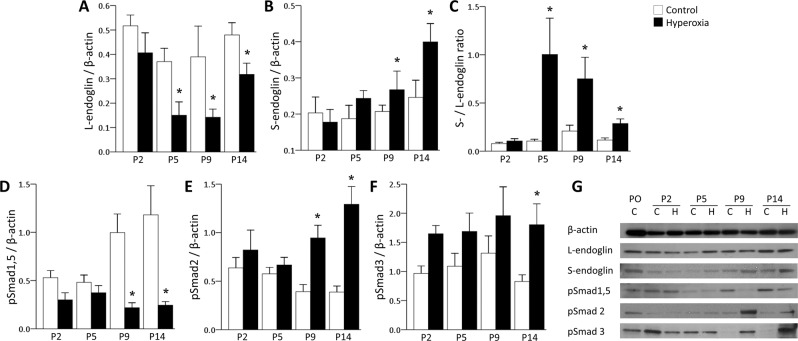
Hyperoxia-induced alterations in L-/S-endoglin and Smad proteins in mice. (**A,B**) The protein level of L-endoglin was significantly decreased at P5, P9 and P14 in the hyperoxia group. In contrast, S-endoglin was significantly increased at P9 and P14 in the hyperoxia group. (**C**) The calculated S-endoglin/L-endoglin ratio was significantly increased at P5, P9 and P14 in the hyperoxia group. (**D–F**) The protein levels of phosphorylated Smad1/5 were significantly decreased in the hyperoxia group, whereas the protein levels of phosphorylated Smad2 and phosphorylated Smad3 were significantly increased in the hyperoxia group compared to the control group at P9 and P14. (**G**) Representative images of western blots in the control (**C**) and hyperoxia (**H**) groups. The data are expressed as the mean ± SEM (n = 4–6 in each group). White bars denote the control group, and black bars denote the hyperoxia group. Groups of data were compared with the corresponding time point by the Mann-Whitney U test. Significant differences (*p* < 0.05) compared with the control group are marked by *.

The protein levels of L-endoglin and phosphorylated Smad1/5 were significantly decreased in the hyperoxia group compared to the control group, while those of S-endoglin and phosphorylated Smad2/3 were significantly increased (Fig. 3A–G), demonstrating that these proteins were oppositely regulated by hyperoxia exposure. The calculated S-/L-endoglin ratio was significantly higher in the hyperoxia group than in the control group (Fig. 3C). Endoglin is an important accessory receptor for TGFβ-mediated signalling, and it is well known that TGFβ type I receptors (ALK1 and ALK5) have very high specificities for Smad phosphorylation (ALK1 phosphorylates Smad1/5, while ALK5 phosphorylates Smad2/3)^23,37,43–45^. Given all these findings, we postulate that hyperoxia exposure increases the production of S-endoglin, resulting in upregulation of Smad2/3 phosphorylation through ALK5.

### Hyperoxia impairs the tubular growth of ECs, while blockade of ALK5 restores normal tubular growth

**Figure 4:**
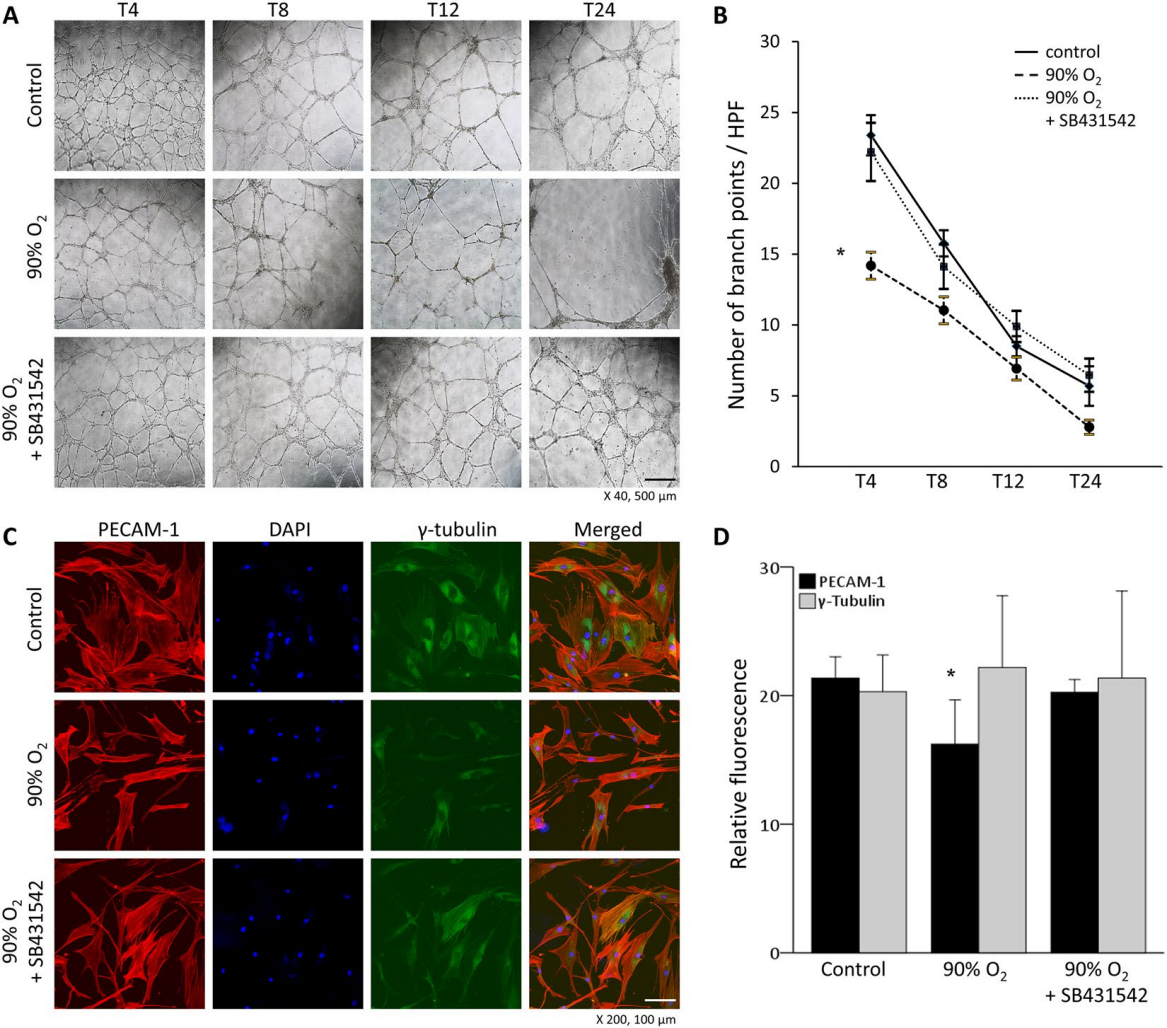
Tubular growth of human pulmonary microvascular endothelial cells (HPMECs). (**A**) Representative light microscopic images of the tubular growth of HPMECs. Magnification 40×. Bars, 500 µm. (**B**) Tube formation assays showed decreased branch points of HPMECs exposed to hyperoxia. The synthetic ALK5 inhibitor SB431542 restored normal tubular growth. (**C**) Immunofluorescent triple staining with antibodies against PECAM-1, DAPI and ɣ-tubulin. Magnification 200×. Bars, 100 µm. (**D**) Relative fluorescence of PECAM-1 was decreased in hyperoxia-exposed HPMECs. SB431542 restored its fluorescence similar to that observed in control HPMECs. There was no difference in the relative fluorescence of ɣ-tubulin. The data are expressed as the mean ± SEM (n = 4–6 in each group). Groups of data were compared with the corresponding time point by the Mann-Whitney U test. Significant differences (*p* < 0.05) compared with the control group are marked by *.

We observed a pronounced decrease in tubular growth and branching in human pulmonary microvascular endothelial cells (HPMECs) exposed to hyperoxia compared to control cells (Fig. 4A,B). To examine the effect of ALK5 blockade on hyperoxia-induced tubular growth impairment, we added the synthetic ALK5 inhibitor SB431542. As shown in Fig. 4A,B,D, ALK5 inhibition restored normal tubular growth and extinguished the effect of hyperoxia on HPMECs. The relative fluorescence effects were more obvious for PECAM-1 than for γ-tubulin (Fig. 4C,D). From the above results, we can hypothesize that the downstream action of ALK5 is involved in hyperoxia-related pulmonary dysangiogenesis.

### Hyperoxia alters the production of L- and S-endoglin and phosphorylated Smad proteins in ECs

**Figure 5:**
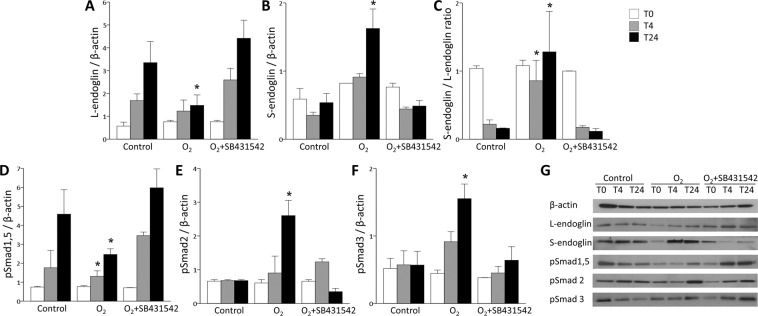
Hyperoxia-induced alterations in L-/S-endoglin and Smad proteins in HPMECs. (**A,B**) The protein level of L-endoglin was significantly decreased in the hyperoxia group. In contrast, S-endoglin was significantly increased in the hyperoxia group at T24. These protein levels were restored by treatment with the SB431542 ALK5 inhibitor. (**C**) The calculated S-endoglin/L-endoglin ratio was significantly increased at T4 and T24 in the hyperoxia group. The balance was restored by SB431542. (**D–F**) The protein levels of phosphorylated Smad1/5 were significantly decreased in the hyperoxia group, whereas the protein levels of phosphorylated Smad2 and phosphorylated Smad3 were significantly increased in the hyperoxia group compared to the control group at T24. These protein levels were restored by treatment with SB431542. (**G**) Representative images of western blots in the control, hyperoxia and hyperoxia + SB431542 groups. The data are expressed as the mean ± SEM (n = 4–6 in each group). White bars denote T0, grey bars indicate T4, and black bars indicate T24 in each group. Groups of data were compared with the corresponding time point by the Mann-Whitney U test. Significant differences (*p* < 0.05) compared with the control group are marked by *.

In hyperoxia-exposed HPMECs compared to control HPMECs, the protein levels of L-endoglin and phosphorylated Smad1/5 were significantly decreased, while the protein levels of S-endoglin and phosphorylated Smad2/3 were significantly increased, which were restored by treatment with the SB431542 ALK5 inhibitor (Fig. 5A–G). The calculated S-/L-endoglin ratio was significantly higher in the hyperoxia group than in the control group, but balance was restored by ALK inhibition (Fig. 5C). Taken together, these results indicate that hyperoxia exposure causes the increased production of S-endoglin in HPMECs, resulting in the upregulation of Smad2/3 phosphorylation, and that these actions are mediated by ALK5. This result also reflects the possibility that immature human pulmonary ECs can synthesize considerable amounts of S-endoglin together with L-endoglin. The absence of an anti-S-endoglin antibody renders it difficult to confirm this reaction as an endogenous protein.

### Ectopic expression of L-or S-endoglin alone does not modify the production of phosphorylated Smads or VEGF, CTGF or PECAM-1 proteins

**Figure 6:**
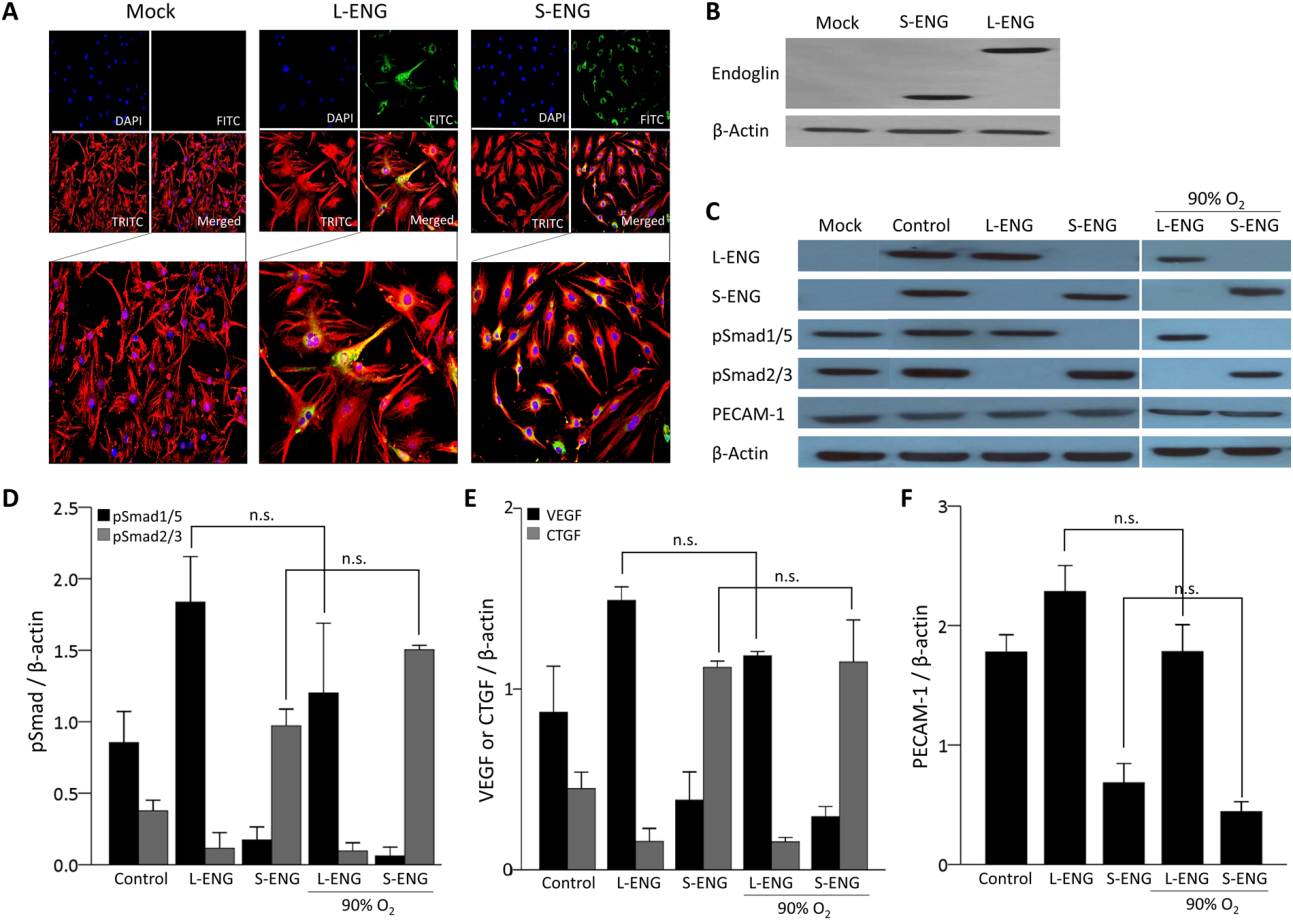
Production of Smad proteins and PECAM-1 in L-/S-endoglin transfected HPMECs. Representative images of (**A**) immunofluorescence and (**B**) western blots of endoglin in mock, L-endoglin (L-ENG)- and S-endoglin (S-ENG)-transfected HPMECs. (**C–F**) Mock, L-ENG- and S-ENG-transfected cells were serum starved for 12 h before 4 h of TGFβ1 treatment. Experimental 90% O_2_ was administered during TGFβ1 treatment. (**D–F**) TGFβ1-induced Smad1/5 phosphorylation was noted only in L-ENG-transfected cells, whereas Smad2/3 phosphorylation was noted only in S-ENG-transfected cells. (**D**) Hyperoxia did not modify Smad phosphorylation or VEGF, CTGF or PECAM-1 production in either L-ENG- or S-ENG-transfected cells. The data are expressed as the mean ± SEM (n = 4–6 in each group). Groups of data were compared by the Mann-Whitney U test. Non-significant differences are expressed as n.s.

The ectopic expression of L- and S-endoglin in HPMECs was assessed by immunofluorescence and western blot analysis. Both L- and S-endoglin were homogeneously distributed on the cell membrane (Fig. 6A). L- and S-endoglin were also detected by western blot analysis (Fig. 6B,C). As we expected, no endoglin expression was observed in mock cells. TGFβ1-induced Smad1/5 phosphorylation was noted only in L-endoglin-transfected cells, whereas Smad2/3 phosphorylation was noted only in S-endoglin-transfected cells (Fig. 6D). VEGF and PECAM-1 expression was markedly augmented in L-endoglin- but not S-endoglin-transfected HPMECs. In contrast, CTGF expression was markedly increased in S-endoglin- but not L-endoglin-transfected HPMECs (Fig. 6E,F). However, hyperoxia did not modify Smad phosphorylation or VEGF, CTGF or PECAM-1 production in either L-endoglin- or S-endoglin-transfected cells (Fig. 6E,F). These results indicate that L- and S-endoglin regulate pulmonary angiogenesis differentially and that these contrasting effects are mediated by ALK1 or ALK5 phosphorylating their specific downstream Smad proteins.

## Discussion

Here, we demonstrated, for the first time, the existence of two endoglin isoforms and an altered balance between these isoforms in hyperoxia-induced BPD models. We found that S-endoglin expression was increased by high oxygen exposure in a murine BPD model and in human pulmonary ECs. This upregulation of S-endoglin led to decreased angiogenesis and alveolar simplification in neonatal murine lungs and inhibited tube formation in a 3D culture of human pulmonary microvascular ECs.

Endoglin (also known as CD105 or TGFβ type III receptor) is not a true receptor for TGFβ, but it regulates the phosphorylation levels of the TGFβ type II receptors, ALK1 and ALK5^47^. Recent studies have reported that downregulation of TGFβ-endoglin-ALK1-Smad1/5 signalling and upregulation of TGFβ-endoglin-ALK5-Smad2/3 signalling play important roles in the developmental arrest of not only pulmonary angiogenesis but also alveolarization^25,42,44,48,49^. However, it is unclear how endoglin determines this pathway. To date, most studies published on endoglin have focused on L-endoglin, although L-endoglin and S-endoglin are co-expressed and distinguishable *in vivo*^32,36,37,39–43,48,50–52^. This finding explains the contradictory results in the quantitative expression of endoglin in experimental BPD models or postmortem BPD patients in which the total quantity of endoglin has been evaluated^9,11,18,25,32,40,42,45,53^.

Two alternatively spliced isoforms of endoglin have been reported as follows: L- and S-endoglin^32,36,37,39–43,48,50–52^. S-endoglin is characterized by the retention of intron 14 in the mature mRNA^34,36,37,39–43^. Because the stop codon is included in the non-spliced intron 14, S-endoglin contains only 14 amino acids in the cytoplasmic domain, while L-endoglin contains 47 amino acids in its intracellular domain^34,39^. These structural differences may account for the distinct functions of L- and S-endoglin^34,37^. Experimental data have demonstrated that the S-endoglin:L-endoglin ratio is increased during senescence and oxidative stress in ECs *in vitro* as well as during ageing in vascularized tissues^37,39,40,54^. The switch between L-endoglin and S-endoglin affects TGFβ-mediated cell signalling towards promoting the ALK5-Smad2/3 pathway instead of the ALK1-Smad1/5 pathway. This phenomenon is thought to be due to the preferential affinity of the short cytoplasmic domain for ALK5^34,36,37,43^.

Several studies have demonstrated that L-endoglin-ALK1-Smad1/5 signalling enhances the migration, proliferation, and tube formation of ECs, while the S-endoglin-ALK5-Smad2/3 pathway inhibits the activity of ECs. In addition, the expression of S-endoglin is increased in senescent ECs, and increased S-endoglin levels are related to endothelial ageing, damage or dysfunction^32,37,54–56^. Unfortunately, little is known about the role of endoglin isoforms in developing lungs and immature pulmonary ECs during the alveolarization period. Therefore, we hypothesized that endoglin may be the key molecule determining the TGFβ signalling pathway and that elevated S-endoglin could be linked to the pathogenesis of BPD.

Indeed, we observed that oxygen exposure increased S-endoglin levels and the S-/L-endoglin ratio in both mouse and pulmonary ECs. According to our results, S-endoglin behaves as an antiangiogenic molecule in contrast to proangiogenic L-endoglin. Elevated Smad2/3 phosphorylation, a marker of ALK5 activity, was also observed with paucity of vessels and angiogenesis accompanied by impaired alveolarization in mice and by tubular growth impairment in pulmonary ECs. Furthermore, the inhibition of ALK5 increased L-endoglin levels and Smad1/5 phosphorylation similar to control cells, with the restoration of normal tubular growth in pulmonary ECs. These data lend support that the unbalanced hyper-expression of S-endoglin alters the balance between TGFβ-ALK1-Smad1/5 and TGFβ-ALK5-Smad2/3 signalling in pulmonary ECs towards ALK5 activation with Smad2/3 phosphorylation and suggest that this process may underlie the impaired pulmonary angiogenesis resulting in disrupted alveolarization in the developing lung. To assess the impact of the two isoforms of endoglin in pulmonary ECs, transfected cells overexpressing human L-endoglin or S-endoglin as well as mock transfectants were investigated. Although this experimental method does not reflect the *in vivo* situation, the results indicate that L- and S-endoglin regulate pulmonary angiogenesis differentially, and these contrasting effects are mediated by ALK1 or ALK5 phosphorylating their specific downstream Smad proteins. We could not analyse the functional effects of S-endoglin silencing. The only difference between S-endoglin and L-endoglin mRNA transcripts is an intronic sequence of 135 bp that is retained in S-endoglin^37^. For this reason, there is no consensus small interfering RNA motif for this small sequence in the current study.

In the context of pulmonary vascular remodelling, the abnormally enhanced S-endoglin expression during exposure to hyperoxia together with ALK5 upregulation we observed are noteworthy, since S-endoglin and ALK5 activities are related to a decreased vasodilatory response and decreased endogenous nitric oxide synthase expression in lungs^23,24,36,37^. This finding means that S-endoglin overexpression may contribute to the development of pulmonary hypertension associated with BPD. In addition, because hyperoxia-exposed ECs did not form neoangiogenic webs on the Matrigel assay and displayed a high S-/L-endoglin ratio in the present study (Fig. 5), we can infer that unbalanced S-endoglin expression may be linked to precocious lung and pulmonary vascular maturation at the expense of developmental potential in preterm infants.

To our knowledge, the present study is the first report to demonstrate the dysregulated expression of two endoglin isoforms in the neonatal lungs and pulmonary microvascular ECs in the context of BPD pathogenesis. Our observations provide supportive proof for the crucial role of endoglin in the TGFβ signalling pathway in the developing lung and suggest a new therapeutic target for prevention or treatment of BPD. Although signalling by S-endoglin may play an important role in hyperoxia-induced BPD models, the association of S-endoglin with other factors contributing to BPD, such as inflammation, glucocorticoids, and mechanical ventilator-induced lung injuries, remains to be determined. In addition, much more needs to be learned about the mechanisms that regulate the production of S-endoglin, including the alternative splicing of endoglin mRNA in premature infants, and the effects of altered S-/L-endoglin balance on the function, growth and maturation of other vascular organs, especially the kidney, pulmonary and systemic vasculatures themselves.

## Methods

### Animals and hyperoxia exposure

All animal studies were performed at Inha University Hospital Biomedical Research Institute. The experimental protocol and procedures were approved by the Institutional Animal Care and Use Committee of Inha University (INHA 170330-488-1). All experiments were performed in accordance with relevant guidelines and regulations. Three-day-old male C57BL/6 J mice were used to examine the saccular and early alveolar stages of lung development in the mouse. Because sex-specific differences are well known in hyperoxic lung injury, we selected only male pups to exclude the bias effect from the sex difference^57,58^. The sex of mice was determined based on the morphological features of external genitalia^59^. In total, 54 mouse pups from 10 pregnant mice were used in the experiment. Animals were randomly divided into two groups as follows: control and hyperoxia groups.

The mice in the hyperoxia group were kept with a dam in a cage within a Plexiglas hyperoxic chamber with a continuous flow of oxygen to reach an oxygen concentration of 70% for 14 days^60^. The mice in the control group were continuously maintained in room air for 14 days. In mice, postnatal day (P) 14 is considered to represent the infant period of humans and the timing of active alveolarization and microvascular maturation^61^. Nursing dams were switched on alternate days between hyperoxic and normoxic cages to prevent oxygen toxicity. The oxygen concentration inside the Plexiglas hyperoxic chamber was continuously monitored using an oxygen sensor (Coy Laboratory Products, Grass Lake, MI, USA). At each time point (P2, P5, P9 and P14), mice were sacrificed. After thoracotomy, 10 to 40 μl (4 μl/g of body weight for each pup) of phosphate buffered saline (PBS) was infused through the trachea. The right lungs of the pups were immediately stored at −70 °C for biochemical analysis, and the left lungs were fixed in 4% paraformaldehyde solution, embedded in paraffin and stored at 4 °C for histological analysis.

### Lung morphometry

Haematoxylin and eosin (H&E)-stained sections were photographed using a digital camera system (Leica DFC 280; Leica, Wentzler, Germany) with a microscope (Olympus BX51; Olympus, Tokyo, Japan) at 100 × magnification. Images were analysed using the morphometric method as described previously^25^. From the distal lung sections, 4 random non-overlapping high-powered fields (HPFs) per pup were used for morphometric evaluations. The mean chord length (L_m_) provided an estimate of the distance from one airspace wall to an adjacent airspace wall^25^. The alveolar SA was calculated using the following equation: SA = 4 × VD_T × _lung volume / L_m_^25^. Tissue volume densities (VD_T_) were determined using a 10 × 10 grid (the grid element side length was approximately 29 μm)^25^. Lung volume was determined by measuring the displacement of water by lungs after fixation^25^. A total of 20 measurements from 5 random areas on 4 random HPFs were performed for each pup^25^.

### Immunohistochemistry of mouse lungs

For the immunohistochemical evaluation of PECAM-1 (CD31), the deparaffinized lung sections were incubated with a PECAM-1 antibody (Millipore, MA, USA) and a biotin-labelled donkey anti-mouse secondary antibody (Jackson ImmunoResearch Laboratories, West Grove, PA). Immune complexes were visualized using 3,3-diaminobenzidine tetrachloride (DakoCytomation, Denmark). Sections were lightly counterstained with haematoxylin, cleared and mounted with mounting medium. Immunostained sections were examined using a camera system with a microscope at 200 × magnification. Three mouse pups were examined per group at each time point. Four random non-overlapping distal lung sections per pup were used and 5 random non-overlapping fields per section were captured for quantitative imaging (Leica DFC 280; Leica, Wentzler, Germany)^25^.

### Real-time RT-PCR

The quality and purity of the isolated total RNA from lung tissues were determined by spectrophotometry at 260 and 280 nm. Single-stranded cDNA was synthesized from 1 μg of RNA using a cDNA synthesis kit (Bio-Rad, Hemel Hempstead, UK) according to the manufacturer’s instructions. The transcripts were amplified, and real-time PCRs were run with SYBR Green Mastermix (Bio-Rad, Hercules, CA, USA). The data were analysed using Opticon Monitor software (Ver 3.1, Bio-Rad). β-Actin was used as the housekeeping gene, and the relative gene expression of L-endoglin, S-endoglin, VEGF and CTGF was calculated. For experiments with mouse lungs, the cDNA was used as a template for real- time PCR performed with specific primers. The mouse primers were as follows: L-endoglin (FW, 5′-GACCTGTCTGGTAAAGGCCTTGTCCTG-3′; RV, 5′-CTGGGGCCACGTGTGTGAGAATAG-3′); S-endoglin (FW, 5′-GACCTGTCTGGTAAAGGCCTTGTCCTG-3′; RV, 5′-CTGAGGGGCGTGGGTGA AGG-3′); VEGF (FW, 5′- AGAAAGCCCTGAAGTGGTG-3′; RV, 5′- ACTCCAGGGCTTCATCATTG-3′); and CTGF (FW, 5′-ACCCAACTATGATTAGAGCC-3′; RV, 5′-TTGCCCTTCTTAATGTTCTC-3′)^43,46,62^.

### Cell culture and hyperoxia exposure

HPMECs (PromoCell, Heidelberg, Germany) isolated from a 60-year-old male Caucasian donor were used at passages 3‒4 and cultured in EC growth medium MV containing 5% foetal calf serum (FCS), 0.4% EC growth supplement (bovine hypothalamic extract), 10 ng/ml recombinant human epidermal growth factor, 1 μg/ml hydrocortisone and 90 μg/ml heparin (PromoCell, Heidelberg, Germany). HPMECs were grown in a humidified incubator containing 5% CO_2_ at 37 °C as recommended by the manufacturer (PromoCell, Heidelberg, Germany). For hyperoxia culture, cells were grown in a multi-gas incubator (APM-30D, ASTEC, Fukuoka, Japan) with regulated delivery of 95% O_2_ and 5% CO_2_ gas.

### Angiogenic tubular formation assay

HPMECs grown to approximately 80% confluence in 6-well plates were silenced. After 24 h of silencing, the cells were detached with TrypLE Express (Invitrogen, CA, USA), and 1 × 10^5^ cells were added to 250 μl of serum-free Matrigel (PromoCell, Heidelberg, Germany), gently mixed, polarized for 30 min at 37 °C, deposited into a 24-well culture plate (Bio-Rad, Redmond, WA, USA) and treated with 100 pM recombinant human TGFβ1 (R&D Systems, Minneapolis, MN, USA). Cells were then covered with 50 μl of complete medium containing 10% FCS and grown at 37 °C under 5% CO_2_ or 90% O_2_ for the indicated time periods (0, 4, 12, 24, and 48 h). To inhibit the ALK5 pathway, the cells were treated with 10 μM SB431542, an inhibitor of Smad2 phosphorylation (R&D Systems, Minneapolis, MN, USA). Angiogenesis was evaluated by counting the number of tube and network formations. Only tubular structures connecting two cell clusters were considered for branch measurements. Representative images were taken using a BX51 microscope (Olympus, Tokyo, Japan) with an attached camera (Leica DFC 280; Leica, Wentzler, Germany) at 40 × magnification. At each time point, cells were processed with Dispase (Bio-Rad, Hercules, CA, USA) for biochemical analysis.

### Immunofluorescence analysis and confocal microscopy

Cells were grown in chambered cover glasses with or without Matrigel for 24 h. The cells were fixed with 2% paraformaldehyde for 20 min at room temperature and permeabilized with 0.5% Triton X-100 in PBS for 10 min at room temperature. The cells were rinsed three times with 100 mM glycine in PBS for 15 min and blocked with TNB [0.1 M Tris-HCl, 0.15 M NaCl, 0.5% blocking reagent (Boehringer Mannheim GmbH, Germany)] plus 10% FBS for 2 h at 37 °C. The cells were then incubated with the indicated antibodies diluted in TNB overnight at room temperature, and after washing with 0.1% NP-40 in PBS, the cells were incubated with goat anti-mouse secondary antibodies (Invitrogen, CA, USA) for 2 h at 37 °C. A goat anti-mouse secondary antibody marked with the red fluorochrome Alexa Fluor 594 (Invitrogen, CA, USA) was used to detect PECAM-1 or ɤ-tubulin. A goat anti-rabbit secondary antibody marked with the green fluorochrome Alexa Fluor 488 (Invitrogen, CA, USA) was used to detect ɤ-tubulin or human influenza haemagglutinin (HA). For nuclear counterstaining, blue-fluorescent DAPI staining was performed (Vector Laboratories, CA, USA). For multiple staining, samples were incubated sequentially with the indicated primary and secondary antibodies. The preparations were analysed with a Leica TCSSP5 (Leica Microsystems, Wentzler, Germany) confocal microscope. The analyses were captured at 200 × magnification. A total of 60 measurements from five-well slides were performed per group.

### Transfection of L- or S-endoglin in HPMECs

The cDNAs encoding full-length human L- and S-endoglin polypeptides were cloned by application of the complete open reading frame (C-ORF) strategy and bioinformatics. L- and S-endoglin containing an N-terminal HA-tag were amplified by RT-PCR using the universal primers CMV 5′- AGAGAGTAGGTACAACGAAGGAGG- 3′ and 5′-ACGTTCTCGAAGGCATCTCTGGAA-3′, and the L- and S-endoglin expression vectors (pcDNA3.1 + /N-HA_L-ENG and pcDNA3.1 + /N-HA_S-ENG) were constructed by cloning the RT-PCR product into the *Bam*HI site of pcDNA3.1 + (Invitrogen, San Diego, CA, USA). Cells were transfected with the expression vectors for 2 h using SuperFect^**®**^ transfection reagent (Qiagen, Valencia, CA, USA) as indicated by the manufacturer. The cells were grown in FBS-free medium for 12 h and treated with TGFβ1 (100 pM) for 4 h at 37 °C under 5% CO_2_ or 90% O_2_. Then, the cells were lysed for western blot analysis. Detection of co-precipitated endoglin protein isoforms in the western blots was performed with anti-HA antibodies.

### Western blot analysis

Lung tissues and cells were homogenized in lysis buffer with protease inhibitor cocktail (Roche, IN, USA)^25^. After determining the protein concentration by DC protein assay kits (Bio-Rad, Hercules, CA, USA), total soluble proteins (20 μg) were separated on 4–12% gradient gels (Invitrogen, CA, USA)^25^. The proteins were transferred to a nitrocellulose membrane (Amersham, Little Chalfont, UK). Nonspecific binding was blocked with 5% skim milk (BD, CA, USA) at room temperature for 1 h^25^. Then, the protein blots were probed with specific primary antibodies (1:1000 dilutions) against phospho-Smad1/5 (Cell Signaling, Frankfurt, Germany), phospho-Smad2 (Millipore, MA, USA), phospho-Smad3 (Millipore, MA, USA), PECAM-1 (Millipore, MA, USA), and L- and S-endoglin (R&D Systems, Minneapolis, MN, USA) overnight at 4 °C. After incubating with host-specific secondary antibodies (goat anti-rabbit IgG-HRP) for 1 h at room temperature, the immunoreaction was detected with a chemiluminescent substrate kit (Thermo Scientific, Rockford, IL, USA) and quantitated by densitometric scanning of the X-ray film with SLB MyImager (UVP Inc, Upland, CA, USA)^25^. The blots were normalized for protein loading by washing in stripping solution and reprobing with a monoclonal antibody against β-actin (Cell Signaling, Frankfurt, Germany)^25^.

### Statistical analysis

Statistical analyses were performed using SPSS version 21.0. The data are expressed as the mean ± standard error of the mean (SEM) of four or more independent experiments. The Mann-Whitney U test was used for comparisons between the hyperoxia and control groups. *P* < 0.05 was considered statistically significant.

## Data Availability

The datasets generated and analysed in the present study are available upon request from the corresponding author.
